# High degree of conservation of the enzymes synthesizing the laminin-binding glycoepitope of α-dystroglycan

**DOI:** 10.1098/rsob.210104

**Published:** 2021-09-29

**Authors:** Maria Giulia Bigotti, Andrea Brancaccio

**Affiliations:** ^1^ School of Translational Health Sciences, Research Floor Level 7, Bristol Royal Infirmary, Upper Maudlin Street, Bristol BS2 8HW, UK; ^2^ School of Biochemistry, University Walk, University of Bristol, Bristol BS8 1TD, UK; ^3^ Institute of Chemical Sciences and Technologies ‘Giulio Natta’ (SCITEC) - CNR, Largo F.Vito 1, 00168, Rome, Italy

**Keywords:** dystroglycan, post-translational glycosylation, M3 core structure, laminin-binding glycoepitope, glycosyltransferases, protein evolution

## Abstract

The dystroglycan (DG) complex plays a pivotal role for the stabilization of muscles in Metazoa. It is formed by two subunits, extracellular α-DG and transmembrane β-DG, originating from a unique precursor via a complex post-translational maturation process. The α-DG subunit is extensively glycosylated in sequential steps by several specific enzymes and employs such glycan scaffold to tightly bind basement membrane molecules. Mutations of several of these enzymes cause an alteration of the carbohydrate structure of α-DG, resulting in severe neuromuscular disorders collectively named dystroglycanopathies. Given the fundamental role played by DG in muscle stability, it is biochemically and clinically relevant to investigate these post-translational modifying enzymes from an evolutionary perspective. A first phylogenetic history of the thirteen enzymes involved in the fabrication of the so-called ‘M3 core’ laminin-binding epitope has been traced by an overall sequence comparison approach, and interesting details on the primordial enzyme set have emerged, as well as substantial conservation in Metazoa. The optimization along with the evolution of a well-conserved enzymatic set responsible for the glycosylation of α-DG indicate the importance of the glycosylation shell in modulating the connection between sarcolemma and surrounding basement membranes to increase skeletal muscle stability, and eventually support movement and locomotion.

## Graphical abstract

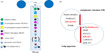

## 1. Background

Dystroglycan (DG) is an extracellular matrix (ECM) protein complex with a wide tissue distribution, that ranges from skeletal, cardiac and smooth muscle to the central and peripheral nervous systems [1,2]. DG is encoded by the *DAG1* gene and translated from a single mRNA as a precursor which undergoes post-translational modifications that include extensive decoration with carbohydrates and processing into two subunits: the highly glycosylated extracellular α-DG and the transmembrane β-DG [3]. The two subunits interact non-covalently to form a bridge between the ECM and the actin cytoskeleton. α-DG acts as a receptor for ECM proteins containing laminin-globular (LG) domains such as laminin and agrin, among others [4]. In previous bioinformatic analyses, we have demonstrated the presence of the DG complex and gene in all the Metazoan lineages starting from Porifera, underlining its considerable biological and pathophysiological importance [5,6].

The carbohydrate moieties of α-DG are mainly concentrated within the elongated central *mucin-like* region that separates two-terminal globular domains [7]. Alterations in the glycosylation shell of α-DG (i.e. hypoglycosylation) can induce muscular dystrophies, referred to as primary or secondary dystroglycanopathies (hereinafter DGpathies), which present themselves with phenotypes that range from minor and later-onset to congenital and severe [8]. In the so-called primary DGpathies, caused by mutations in *DAG1*, α-DG glycosylation and/or the overall stability of the DG polypeptide can be affected [9]. The significance of DG integrity is stressed by the evidence that *DAG1* knockout in mice is lethal during gestation, as early as day E6.5 [10], while a mutation that abolishes the entire complex and leads to post-natal mortality was found in a human family [11].

Glycosylation is one of the most important and ubiquitous forms of post-translational modification and can potentially affect the correct targeting, trafficking and sorting of a protein, thus influencing its stability and function [12]. Therefore, it is probably not a mere coincidence that the series of neuromuscular diseases collectively defined as secondary DGpathies are caused by mutations in genes coding for proteins involved in the *O*-glycosylation of α-DG [8,9]. Indeed, α-DG is extensively glycosylated with *N*-linked as well as *O*-linked groups [13]. *O*-glycosylation of α-DG follows one of the multiple *O*-mannosylation pathways found in eukaryotes [14]. Namely, it features the modification of some specific Thr residues, like for example Thr 317 and 319 [15–17] within the central mucin-like region of α-DG [5,7].

The α-DG *core protein* is heterogeneously glycosylated, and this is particularly evident in its skeletal muscle isoform [13]. In fact, the presence of a mixed population of sugars causes a characteristic ‘blurred’ appearance of the α-DG band in Western blots, indicating a sort of Gaussian distribution of molecular masses that in skeletal muscle is typically centred around 156 kDa [13]. Accordingly, a series of different ‘core carbohydrate structures' have been detected, namely the M1, M2 and M3 core structures [17]. The M3 core structure starts to be formed readily in the endoplasmic reticulum (ER) and subsequently elongated in the Golgi apparatus, while M1 and M2 are generated in the *cis*-Golgi, upon transport from the ER, by the action of POMGnT1 and other enzymes [18].

The heterogeneous distribution of molecular masses observed in α-DG might depend on variability in (i) the number of M1/M2 or (ii) of M3 core structures for each α-DG molecule, respectively, or in (iii) the length of each M3 core structure, and/or on differential contributions arising from each of the aforementioned aspects.

The M3 core structures are the most crucial as they include the specific glycoepitope(s) recognized by extracellular binding partners within the basement membranes surrounding skeletal muscle and other tissues [3].

**Figure 1.**
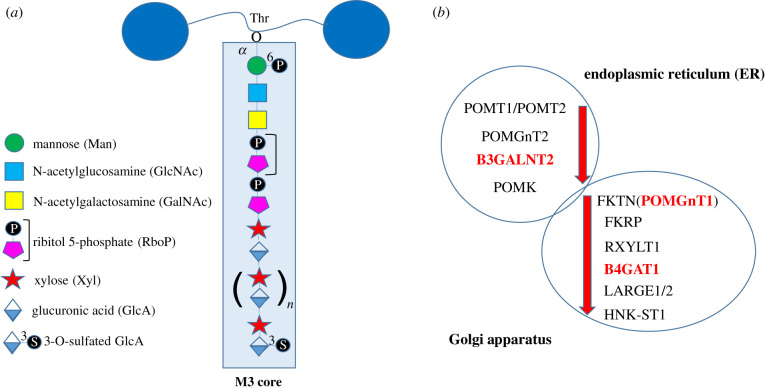
A scheme of the M3 core carbohydrate chain and its specific carbohydrate blocks (*a*) and of the progressive enzymatic cascades present in the ER and Golgi apparatus (*b*). All the relevant details on the consecutive enzymatic steps are reported in table 1. Reported in red in (*b*) are the three enzymes whose orthologues are present in all the lineages analysed.

Each mature α-DG molecule carries numerous (probably 20 to 30) M1 and M2 core structures and several of the Ser/Thr residues within the central mucin-like domain can be found modified with these simpler structures [17,19]. Conversely, the more complex M3 core structures represent just a minority and are found attached to as few as a couple of Thr residues in each α-DG molecule, as suggested for Thr 317 and 319 [16] or Thr 317 and 379 [17], which are highly conserved in vertebrates [5,16]. In mammals, a consensus sequence for *O*-mannosylation (IXPT(P/X)TXPXXXXPTX(T/X)XX) has been identified *via* extensive mass spectrometry analysis and found exclusively in α-DG [15]. The M3 core structure includes a long tandemly repeated polymer of several disaccharide units (xylose-glucuronic acid), defined as matriglycan [20] (figure 1*a*). The length of matriglycan can be variable and depends on the differential activity of LARGE1 (i.e. the enzyme directly responsible for its elongation) and HNK-1ST, which mediates sulfation at the 3-hydroxyl of the terminal glucuronic acid, thus controlling the extent and length of the overall reaction. Such competing action of the two enzymes eventually influences the resulting affinity towards laminin and other α-DG binding partners in different tissues [21]. A further complexity in the control of matriglycan synthesis emerged with the recent finding that also the kinase POMK is needed to regulate the LARGE1-mediated elongation of matriglycan [22].

**Table 1.** Enzymes synthesizing the carbohydrate epitope of α-dystroglycan in the ER and Golgi apparatus, ordered by their reaction sequence, starting from phosphotrisaccharide (M3 core) formation.

code	official name	reaction product added to the chain (or function played)	donor substrate
POMT1	protein *O*-mannosyltransferase 1	mannose (Man)	dolichol monophosphate mannose (Dol-P-Man)^a^
POMT2	protein *O*-mannosyltransferase 2	mannose (Man)	dolichol monophosphate mannose (Dol-P-Man)^a^
POMGnT2	protein *O*-linked mannose *N*-acetylglucosaminyltransferase 2 (beta 1,4-)	N-acetylglucosamine (GlcNAc)	UDP-GlcNAc
B3GALNT2	beta-1,3-*N*-acetylgalactosaminyltransferase 2	N-acetylgalactosamine (GalNAc)	UDP-GalNAc
POMK	protein *O*-mannose kinase	phosphate at C6 position of O-mannose	ATP
FKTN	fukutin	ribitol 5-phosphate (RboP)	CDP-ribitol^b^
POMGnT1	protein *O*-linked mannose *N*-acetylglucosaminyltransferase 1 (beta 1,2-)	binds to the growing saccharide chain and FKTN via its stem domain	—
FKRP	fukutin-related protein	ribitol 5-phosphate (RboP)	CDP-ribitol^b^
RXYLT1	ribitol xylosyltransferase 1	xylose (Xyl)	UDP-Xyl
B4GAT1	beta-1,4-glucuronyltransferase 1	glucuronic acid (GlcA)	UDP-GlcA
LARGE1	LARGE xylosyl- and glucuronyltransferase 1	[Xyl-GlcA]_n_	UDP-Xyl, UDP-GlcA
LARGE2	LARGE xylosyl- and glucuronyltransferase 2	[Xyl-GlcA]_n_	UDP-Xyl, UDP-GlcA
HNK-1ST	sulfotransferase or CHST10 carbohydrate sulfotransferase 10	sulfate at C3 position of terminal GlcA	3′-phosphoadenosine 5′-phosphosulfate (PAPS)

^a^Provided in the cytosol by Dol-P-Man biosynthesis pathway including DPM1/2/3 (belonging to the dolichol-phosphate mannosyltransferase (DPM) Complex) and DOLK (dolichol kinase). Mutations in the gene encoding GDP-mannose pyrophosphorylase B (*GMPPB*), which functions in GDP-Man formation from Man-1-phosphate and GTP, were also reported in DGpathy patients [32].

^b^Provided in the cytosol by ISPD (isoprenoid synthase domain containing) that uses CTP and RboP (produced by an unknown enzyme) as substrates. ISPD is a cyitidyltransferase or CDP-ribitol pyrophosphorylase [33].

A loss, or a reduction, of the O-mannose-bound matriglycan on α-DG can lead to a diminished cell adhesion to the ECM, with pathological consequences. Indeed, the matriglycan scaffold has a crucial physiological value since it represents the site recognized with high affinity by α-DG-binding partners [4]. The degree of overall glycosylation can vary in different tissues and might influence the affinity of α-DG towards its binding partners in a subtle and not yet fully understood way [23].

*O*-glycosylation is a highly controlled process that takes place in the ER/Golgi compartments and mutations in enzymes, such as TRAPPC11 and GOSR2, that are involved in localizing proteins to the Golgi compartment, have been also linked to hypoglycosylation of α-DG and muscular dystrophies [24]. In cells defective for subunits of the conserved oligomeric Golgi (COG) complex, which ‘supervises’ the proper glycosylation of proteins, an impaired glycosylation of α-DG and a consequent susceptibility to proteases [25] have been observed, reminiscent of what reported for the ‘naked’ mucin-like domain expressed in bacterial cells [26].

Aside from these general chaperones, fabricating the core M3 glycoepitope of α-DG requires a large and specific orchestra of enzymatic players, localized in the ER first and then in the *cis*- and *median*-Golgi compartments (figure 1*b*). A great deal of outstanding work conducted in different laboratories has revealed that in mammals there are (at least) 13 enzymes responsible for the consecutive steps leading to the mature M3 core structure [27–30]. Such a high degree of complexity highlights the importance of understanding the molecular mechanism of α-DG glycosylation for potential therapeutic applications [31].

The enzyme full names, acronyms and reaction details are reported in table 1 (see also figure 1). The next three sections describe the sequential glycosylation steps and some additional details, including the relevant human pathologies related to these enzymes that underline their functional importance in mammals.

### 1.1. The M3 O-mannose core structure is formed in the endoplasmic reticulum at specific Thr residues (enzymes involved: POMT1/2, POMGnT2, B3GALNT2 and POMK)

The initial mannosylation reaction of α-DG is carried out by POMT, with POMT1 and POMT2 both needed for protein O-mannosyltransferase activity [34,35]. Site mapping studies have identified only two positions on α-DG for mannosylation by POMT1/2 to originate M3 core structures, namely Thr-317 and Thr-379, although some evidence suggests 319 and 381 may also be sites of M3 modification [17].

Mutations in these enzymes can cause autosomal recessive limb-girdle muscular dystrophies, LGMD2 K and LGMD2N [36], or severe Walker–Warburg syndrome (WWS), which is an autosomal recessive condition characterized by congenital muscular dystrophy, structural brain defects and eye malformations [34,35].

POMGnT2 (but not POMGnT1) is then responsible for starting the elongation of M3 in the ER [37], via the addition of *β*4 GlcNAc to the nascent sugar chain [17,27]. From a spatial-temporal perspective, the O-Man-modified α-DG precursor molecule encounters POMGnT2 in the ER first and POMGnT1 only later in the *cis*-Golgi, where its preferential activity leads to the formation of M1 O-mannose glycans [19] (see below). This points to POMGNT2 possessing specific substrate selectivity beyond simple recognition of an O-Man-modified amino acid, that univocally results in the formation of the M3 core structure [17]. Some of the mutations of POMGnT2 cause severe WWS [38], but others only result in mild forms of limb-girdle muscular dystrophy [39,40], while POMGnT2 knockout mice represent models for cobblestone lissencephaly [41].

The subsequent *β*3 addition of GalNAc to the growing chain is carried out in the ER by B3GALNT2 [42]. However, B3GALNT2 activity does not seem to fully correlate with the severity of the observed muscular dystrophy phenotypes [43]. The last step of this ER enzymatic phase is carried out by POMK (SGK196), a kinase that works with ATP as a donor substrate and is involved in the phosphorylation of the M3 glycan mannose on its C-6 position [44]. Three-dimensional structures of POMK have been solved [45,46], and it was recently shown that LARGE1 (see paragraph 1.3) can work efficiently only when POMK is active [22].

### 1.2. A single tandem of ribitol-phosphates is added in the Golgi (enzymes involved: FKTN, POMGnT1 and FKRP)

Fukutin (FKTN) catalyses the transfer of ribitol-phosphate (RboP) to α-DG using cytidine diphosphate ribitol (CDP-Rbo) as substrate. Among the enzymes of the pathway, FKTN is one of the most extensively characterized biophysically, despite the lack of high-resolution three-dimensional structural data [47–49]. Mutations in the FKTN gene can cause autosomal recessive limb-girdle muscular dystrophy (LGMD2M) [50], while a Dandy–Walker malformation was observed in the cerebellum [51]. The conditional knockout of FKTN in the mouse heart leads to a pathology that is observed only in later adulthood, when a severe cardiac dysfunction emerges [52]. It is worth to note that some missense mutations can instead lead to more severe consequences, such as a lethal case of WWS [53].

Interestingly and quite uniquely, POMGnT1 acts as a sort of ‘enzymatic chaperone’ in the fabrication of the M3 core structure. POMGnT1 binds FKTN via its stem domain and then facilitates the enzymatic action (i.e. addition of RboP) of FKTN on the developing M3 core [18,54,55]. POMGnT1 is localized in the *cis*-Golgi via interaction with the Golgi phosphoprotein-3, GOLPH3 [56], and if it were not thus tethered to the Golgi apparatus, it would probably interfere with the ER-localized enzymatic action of POMGnT2. As a consequence, α-DG would then display a wrong pattern of glycosylation as far as the location (i.e. at the level of specific Thr residues) of the resulting M3 and M1 core structures are concerned. In fact, POMGnT1 has been appropriately defined as a ‘cross-core enzyme’ since it is responsible for fabricating, together with the glycosyltransferases MGAT5B/GnT-IX (Vb), the M1 and M2 core structures [17]. Human α-DG has at least 25 O-mannosylation sites, the majority of which are populated by core M1 and M2 glycan structures via the action of POMGnT1 (M1) followed by MGAT5B/GnT-IX (Vb) (M2). POMGnT1 knockout in mice causes a severe form of muscle–eye–brain diseases [57] as described in certain patients [58], although some specific mutations can cause the less severe autosomal recessive limb-girdle muscular dystrophy, LGMD20 [59].

Like FKTN, FKTN-related protein (FKRP) uses CDP-Rbo as substrate (table 1) and transfers a second consecutive RboP to α-DG [27,28,60]. The synergic activity of these two enzymes produces the tandem RboP unit (RboP–RboP) required for the subsequent synthesis, mediated by RXYLT1, B4GAT1 and LARGE, of the laminin-binding glycoepitope on the O-mannosyl glycan. Mutations of FKRP can cause autosomal recessive limb-girdle muscular dystrophy LGMD2I [61,62].

### 1.3. Matriglycan, the polymer that is recognized by the α-DG binding partners (enzymes involved: RXYLT1, B4GAT1, LARGE1/2 and HNK-1ST)

The matriglycan represents the laminin-binding glycoepitope recognized by laminin and other binding partners [4,20]. Upon the addition of the RboP dimer and prior to the synthesis of the matriglycan polymer, a dimer of xylole and glucuronic acid is added by RXYLT1, a ribitol xylosyltransferase 1 (ribitol *β*1,4-Xylosyltransferase) [63] and B4GAT1 (β-1,4-glucuronyltransferase 1) formerly known as B3GNT1 [64,65]. Then, LARGE1 adds the repeating disaccharide unit [-3Xyl-α1,3GlcA*β*1-]_n_ [66] by employing its double capacity as a xylosyltransferase (domain 1) and a glucuronyltransferase (domain 2) [67]: the tandemly repeated polymer of these disaccharide units thus generated is called matriglycan, [20]. The N-terminal domain of α-DG binds LARGE and it could act as a chaperone for directing the enzymatic activity of LARGE towards its own mucin-like domain [68,69]. LARGE2 is a paralogue of LARGE1 found in Vertebrates [70]. The elongation process ends with the 3-O-sulfation of the last glucuronic acid within matriglycan catalysed by the HNK-1ST sulfotransferase [71] that transfers a sulfate group to the non-reducing end GlcA of matriglycan and prevents extension by LARGE1 [21].

Given the role played by α-DG for muscle stability and muscular dystrophies, we believe that it is important to study the evolutionary implications of this set of enzymes that is crucially responsible for producing the glycoepitope at the basis of α-DG function. In addition, we believe it is equalto trace back the origin of the enzymatic machinery that produces the M3 glycoepitope because the carbohydrate moieties of α-DG are hijacked by some viruses to enter eukaryotic cells [72,73].

## 2. Methods

### 2.1. Sequence comparison and bioinformatic methods

All the details referring to the human sequences used as ‘baits’ to find orthologous sequences of the 13 enzymes in the different animal lineages are reported in table 1. The ‘BLAST’ (Basic Local Alignment Research Tool) resource (specifically blastp, protein–protein BLAST), freely available at NCBI, has been used for all the searches, except that for *Oscarella carmela* belonging to the Homoscleromorpha sponges (Porifera), where the one available at Compagen (www.compagen.org) has been employed. Some additional sequence comparison searches have been performed at the web resource Wormbase (https://wormbase.org) for *Caenhorabditis elegans*. Electronic supplementary material is presented in a single file and includes all the sequence comparison details with the best scores found, enzyme by enzyme and group by group. The protein alignments have been carried out in the multiple sequence alignment resource muscle available at EMBL-EBI (https://www.ebi.ac.uk).

Given the genetic importance of the fruit fly, *Drosophila melanogaster* (Insecta, Diptera), for the study of muscle degeneration (see Results and discussion), some relevant orthologues are reported for Drosophilidae. Both for their genetic and evolutionary importance as well as for their wide use as model organisms, orthologues are also reported for the starlet sea anemone, *Nematostella vectensis* (Cnidaria, Anthozoa), for the *Caenorhabditis* genus which includes the widely studied worm *C. elegans*, for zebrafish, *Danio rerio* (Teleostei, Actinopterygii), and for the house mouse*, Mus musculus* (Mammalia, Rodentia).

## 3. Results and discussion

### 3.1. Homology of orthologous enzymes with their human counterparts

*O*-mannosylation, that was originally discovered in fungi [74,75], is known to be conserved from bacteria to humans [76] and multiple O-man glycosylation pathways have been found in eukaryotes [14]. *O*-mannosylation as such is not characteristic of plants [14], nevertheless very good hits with POMT1 and POMT2 have been found in *Quercus suber (*XP_023912063.1*)*, *Carpinus fangiana* (KAB8356666.1) or *Rhodamnia argentea* (XP_030536296.1). The most likely hypothesis is that such hits originate from contamination with some pathogenic fungi when the plant sequence analysis was performed and deposited in the data bank [77] or, less probably, could represent cases of horizontal gene transfer (HGT) [78]. Indeed, the hit found in *Quercus saber* for example (XP_023912063, 784 aa) is practically identical to a glycosyltransferase from the fungus *Baudoinia panamericana* (XP_007679195, 791 aa, 83% identity on a 100% query cover), and many other very good matches are found with similar sequences belonging to other species of fungi, strongly suggesting a ‘cross-contamination’ of fungi sequences into plant sequences within the NCBI databank.

**Figure 2.**
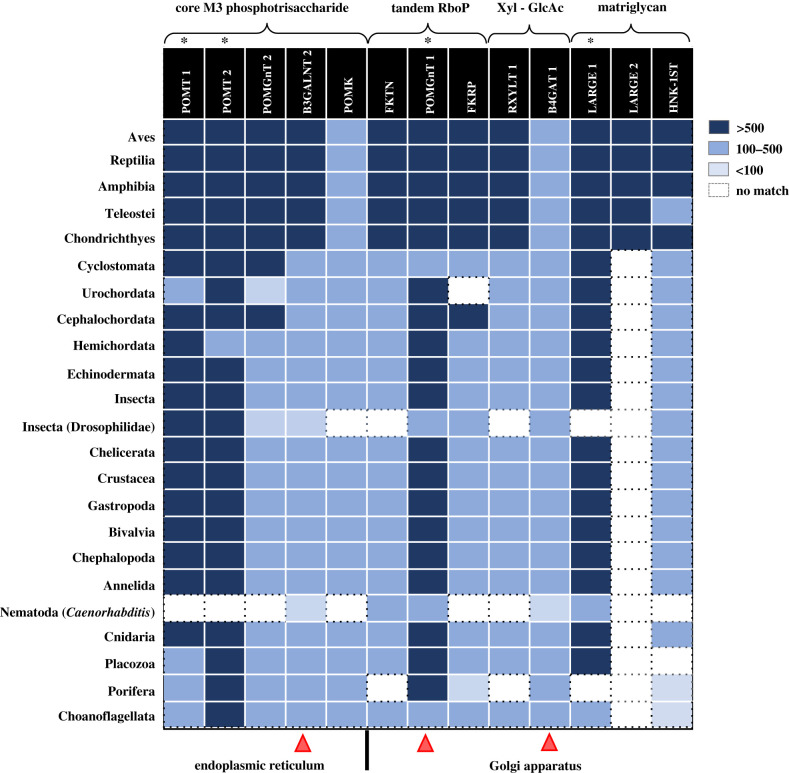
Animal groups versus enzymes box showing the relevant similarities found between the human orthologous sequence baits for the thirteen enzymes (see table 2 for details) and the different animal lineages. The score reported (see electronic supplementary material for the exact values) is the ‘Max score’, i.e. the highest alignment score (bit-score) between the query sequence and the sequence segment found within the database. Score code: greater than 500, navy blue; 100–500, blue; less than 100, pale blue; and no significant match, white. For the matching details (including sequences codes and scores) between the human orthologues and the various groups, see electronic supplementary material. The asterisks mark the four enzymes that are more conserved (POMT1, POMT2, POMGnT1 and LARGE1) while the red triangles highlight B3GALNT2, POMGnT1 and B4GAT1, for which orthologues are present in all the lineages analysed.

The sequences identified in free-living unicellular and colonial flagellate eukaryotic Choanoflagellata and the other metazoan lineages (all displaying with one of the best total scores) have been reported in the electronic supplementary material. All the enzymes (except LARGE2 that originates from a duplication first observed in Chondrichthyes; figure 2; see below) pre-date DG because they are all present in the nearest free-living single-celled eukaryotic precursor of Metazoa, Choanoflagellata where DG is not present [5]. Among basal Metazoa, in the ‘static’ Porifera, some of the 13 enzymes are not present (figure 2). An even higher degree of divergence and loss of significant matching sequences has been observed in the *Caenorhabditis* genus of Nematoda (in which only 5/13 orthologues are found). With the above exception, the enzymes presence is remarkably consistent starting from other basal metazoan, like in basal Placozoa where all the enzymes have been identified (except the sulfotransferase HNK-ST1 and LARGE2) (11/13). It is worth of note that HNK-ST1 was first found in another basal metazoan group such as Cnidaria (12/13). From Annelida upwards, all the enzymes are present except LARGE2. In Urochordata, POMGnT2 and FKRP ‘disappear’, and it remains unclear whether or not there is a paralogue of LARGE1 (figure 2; electronic supplementary material) as reported below in paragraph 3.3.

**Table 2.** Details of the human enzymes used as baits to identify orthologues in the different animal lineages by sequence comparison.

name	location	chr.	length (a.a.)	code
POMT1^a^	ER	9	747	Q9Y6A1
POMT2^a^	ER	14	750	Q9UKY4
POMGnT2	ER	3	580	Q8NAT1
B3GALNT2	ER	1	500	Q8NCR0
POMK	ER	8	350	Q9H5K3
FKTN	CGC/MGC	9	461	O75072
POMGnT1^a^	CGC/MGC	1	660	Q8WZA1
FKRP	CGC/MGC	19	495	Q9H9S5
RXYLT1	Golgi	12	443	Q9Y2B1
B4GAT1	Golgi	11	415	O43505
LARGE1	Golgi	22	756	O95461
LARGE2	Golgi	11	721	Q8N3Y3
HNK-1ST^b^	Golgi	2	356	O43529

^a^involved also in the formation of core M1 and M2.

^b^involved also in the formation of core M1. chr.: chromosome location of the gene. CGC: *cis*-Golgi compartment, MGC: *median*-Golgi compartment.

### 3.2. Conservation and specificity of the M3 core enzymatic cascade

The most conserved enzymes with respect to the human ‘bait’ sequences used in this analysis (table 2) are POMT1/POMT2, POMGnT1 and LARGE1. It is perhaps not a coincidence that some of the more serious forms of dystroglycanopathy observed in human patients (displaying WWS or muscle–eye–brain disease) originate from mutations in some of these enzymes. This seems to be in line with what observed in mouse *knockout* model systems. For example, mutations in human POMGnT1 cause LGMD2O (late childhood, progression moderate) while mice are viable but affected by a severe form of muscle–eye–brain disease [57]. On the other hand, POMGnT2 *knockout* mice present with severe multiple phenotypes during embryonic development and perinatal death. [41,79].

Irrespective of the overall degree of conservation, our analysis shows how POMGnT1, B3GALNT2 and B4GAT1 are the only enzymes always present in all the groups considered (figure 2). It is important to remind that POMGnT1, along with POMT1 and many other known and still unidentified enzymes, is also involved in the formation of the abundant M1/M2 cores [17,19,44,80].

Have the enzymes responsible for the synthesis of the carbohydrate structure including M3, the two RboP, the additional Xyl-GlAc and sulfated Matriglycan evolved specifically for the glycosylation of α-DG? The most obvious answer to this question is no, as the full set of enzymes displaying a relevant degree of conservation to their orthologous human sequences is present in the Choanoflagellata (figure 2), which are single-cell eukaryotes precursors of multicellular organisms where DG cannot be found [5]. Therefore, a possible scenario is that the novel DG must have ‘hijacked’ an ancient pool of already available set of enzymes to fulfill its own ‘sugar decorating purposes'. A related question arises. Is the ‘M3 epitope’ shared by other proteins? In other words, do these enzymes have substrates other than α-DG? Although this remains to be established, α-DG is the only O-mannosylated protein that has been extensively studied so far [80]; therefore, the possible existence of other proteins containing very similar M3 core structures cannot be ruled out. Recently, it has been reported that indeed FKRP directs sialylation of fibronectin at the muscle basement membrane, influencing the overall stability of sarcolemma in muscular dystrophy and therefore strongly suggesting the existence of multiple target/pathways, alternative to DG, affected by the ‘M3’ enzymes [81]

The very high degree of conservation observed in Chordata strengthens the evidence that these 13 enzymes are important for muscle stability (figure 2). All considered, such remarkable conservation of the enzymatic cascade underlies the overall importance of α-DG carbohydrates for its functional activity. The emergence of the full pathway in early metazoan groups such as Placozoa and Cnidaria (well before the rise of more complex bilaterians) may suggest the crucial importance of a fully functional α-DG for stronger connections between the cytoskeleton and the muscular fibres that are needed for a general improvement in movement and locomotion, as already observed in Annelida and Mollusca (especially in Cephalopoda).

In Porifera, the overall degree of conservation is low. This raises the question of the possible existence of an ‘inverse correlation’ between the conservation of an enzymatic cascade that is fundamental for muscle stability and muscle-driven locomotion skills, and the staticity of Porifera in their adult stage. In fact, most sponges have a biphasic life cycle, with a planktonic phase (as moving larvae) and a benthic, less mobile one. Although it might sound like a fascinating hypothesis, it remains unclear if the high degree of divergence observed in sponges is directly related to their life cycle aspects. DG is already present in sponges, but both its degree of glycosylation and its molecular structure are currently scarcely characterized. Indeed, the presence of a region rich in Ser and Thr could have represented an ancestral mucin-like region in DG from Porifera, and mucin-like regions, although much shorter than in vertebrates, are already present in Placozoa and Cnidaria [5]. In Metazoa, laminins started to be present from Porifera and therefore represent binding partners for the α-DG carbohydrate scaffold, leading to the establishment of the high-affinity axis laminin (ECM)—DG sugar platform (membrane)—cytoskeleton that proved to be extremely successful from an evolutionary point of view. As a matter of fact, in the majority of metazoan groups (starting from Mollusca and Annelida), there is a consolidated presence of all the DG-binding partners at the cellular–matrix interface. Aside from detecting conserved orthologues of the relevant enzymes involved, a great deal of biochemical and structural work is granted to elucidate the exact carbohydrate moieties present in the orthologous DG complexes in the various invertebrate lineages [82].

Although the overall degree of glycosylation of α-DG in Porifera remains unclear, several enzymes of the glycosylation cascade are lost (4/13). As already stated, adult Porifera are static organisms and this has possibly favoured an hypoglycosylated α-DG, whereby the development of a specific multi-enzymatic cascade leading to the high level of α-DG glycosylation necessary to confer the muscle stability that movement requires was not needed. However, one necessary disclaimer is that a relatively low amount of sequence information is available for Porifera (i.e. there is still a limited variability of species sequenced) as well as for Placozoa, a group that includes only three genera (*Trichoplax adhaerens*, *Hoilungia hongkongensis* and *Polyplacotoma mediterranea*, with all the current sequence information available only for the former, see electronic supplementary material) and therefore caution is needed when drawing ultimate conclusions for these groups.

Important proteomic work from the Shcherbata's laboratory on *Drosophila*'s DG corroborated and outlined the importance of the DG axis for muscle stability and neuronal development in the fruit fly, underlining its crucial role as a model system for the study of muscular dystrophies as well [83–86]. Still, it is very interesting to note that while in the class Insecta, the cascade appears to be well conserved, some relevant divergences seem to emerge in the family Drosophilidae (order Diptera). For example, while POMT1 and POMT2 have strong orthologues, whose mutations lead to the well-characterized *twisted abdomen* phenotypes [87,88], POMK, FKTN and RXYLT1 do not appear to have orthologues, and POMGnT2 does show only a limited similarity, with the EGF domain-specific O-linked N-acetylglucosamine transferase (a match with a score less than 100 was found for *Drosophila grimshawi*, XP_001989043). Moreover, also the widely and well-conserved B3GALNT2 and POMGnT1 are much less conserved in Drosophilidae as compared to other insect species. In addition, the same match found with B4GAT1/beta-1,4-glucuronyltransferase 1 was identified when using human LARGE1 and LARGE2 as bait (all the sequences details and alignment scores can be found in the electronic supplementary material). Together these observations suggest that there might be some differences within the M3 glycoepitope of *Drosophila*'s DG relative to other Insecta [89], a fascinating hypothesis that awaits targeted experimental work in order to be verified.

### 3.3. Suppression of the M3 enzymatic cascade: a remarkable evolutionary divergence is apparent in the *Caenorhabditis* genus

Nematoda (roundworms) are known to encompass several parasite genera recognized as pathogens for plants and animals (including vertebrates and humans). Indeed, gene loss events frequently derive from a parasitic lifestyle. Interestingly, a DG orthologue is present in the well-characterized and non-parasitic nematode genus *Caenorhabditis* [90], although no extensive α-DG glycosylation shell has been found [5]. Strikingly, in this genus, most of the ‘M3 core’ enzymes are lost (only 5/13 identified; figure 2).

As far as the M3 enzymatic cascade is concerned, the *Caenorhabditis* genus seems to represent an extreme case of evolutionary divergence. This analysis revealed that most of the glycosylating enzymes do not have orthologues in the genus (only 5/13 are conserved, see below). Therefore, it seems unlikely that a ‘full and conventional’ M3 epitope is ever synthesized in those animals. Most relevantly, POMT1 and POMT2 have not been identified (like in plants) and therefore the required initial *O*-mannosylation step cannot take place.

Since the M3 enzymatic cascade is impaired, POMGnT1 and LARGE1 must be involved in other relevant glycosylation pathways. Consequently, it remains highly unclear what kind of carbohydrates (if any) would be present on the DG orthologous protein, also considering the evidence, emerged from our previous analysis, that the central ‘mucin-like’ highly glycosylated domain is missing in *Caenorabditis* [5,6]. As a matter of fact, the orthologue of DG we identified (i) does not share a mucin-like region and (ii) does not have, within its N-terminal region, the conserved S6 domain believed to be important for interacting with LARGE1 during the maturation of α-DG in the Golgi [68,69]. Interestingly, the knockout of the *DAG1* orthologue in *C.elegans* gives rise to a phenotype in the vulvar epithelia as well as in the excretory cell epithelia and in motoneuron axon guidance, but does not seem to affect muscle or be dependent on dystrophin [91].

In members belonging to the *Caenorhabditis* genus, there are only orthologues for B3GALNT2, FKTN, POMGnT1, B4GALT1 and LARGE1. Based on the matches found using the human bait sequences (see details in the electronic supplementary material), it is possible to find the respective five orthologous genes of *C. elegans* (*sqv-2*, *T07A5.1*, *M70.4a*, *bgnt-1.2* and *lge-1*). M70.4a is an orthologue of human FAM3A (FAM3 metabolism-regulating signalling molecule A) and FAM3C (FAM3 metabolism-regulating signalling molecule C) that harbour a domain that was recently shown to belong to POMGnT1 [55]. *Sqv-2* and *sqv-6* have been shown to correspond to human galactosyltransferases (*sqv-2*) and xylosyltransferases (*sqv-6*; although not similar to *RXYLT1*) [92].

It is interesting to note that these enzymes belong to a Golgi apparatus enzymatic machinery involved in the production of glycosaminoglycan chains (starting from xylose) and that *sqv-2* and *sqv-6* mutants display also a vulvar phenotype similar to that observed with the knockout of the DG-like gene of *C. elegans* that does not show a phenotype in muscle [91,92]. In Nematoda, there are no POMT1/POMT2 and O-mannosylation does not seem to be present; however, it is not known whether *C. elegans* DG could carry xylol-based carbohydrate chains instead. In general, these evidence point towards a slightly different role of the DG complex in Nematoda and to a different glycosylation pattern that would match the poorly conserved pattern of enzymes involved in the M3 core synthesis.

### 3.4. *LARGE1* duplication in vertebrates

Contrary to what observed for other genes of the cascade that were already found duplicated ancestrally (POMT1/POMT2 in Choanoflagellata for example), the *LARGE1* gene underwent a duplication event way more recently [70]. Genome duplication events contributed to increase the plasticity and complexity of vertebrate genomes, as observed especially in fish [93,94]. It was proposed that the redundancy in gene repertoires possessed by all vertebrates, including cyclostomes belonging to the agnathans (jawless fishes) lineage, was introduced primarily by two rounds of whole-genome duplications taking place during chordate evolution after the split of the Urochordata and Cephalochordata lineages, but before the radiation of gnathostomes (jawed vertebrates) [95].

**Figure 3.**
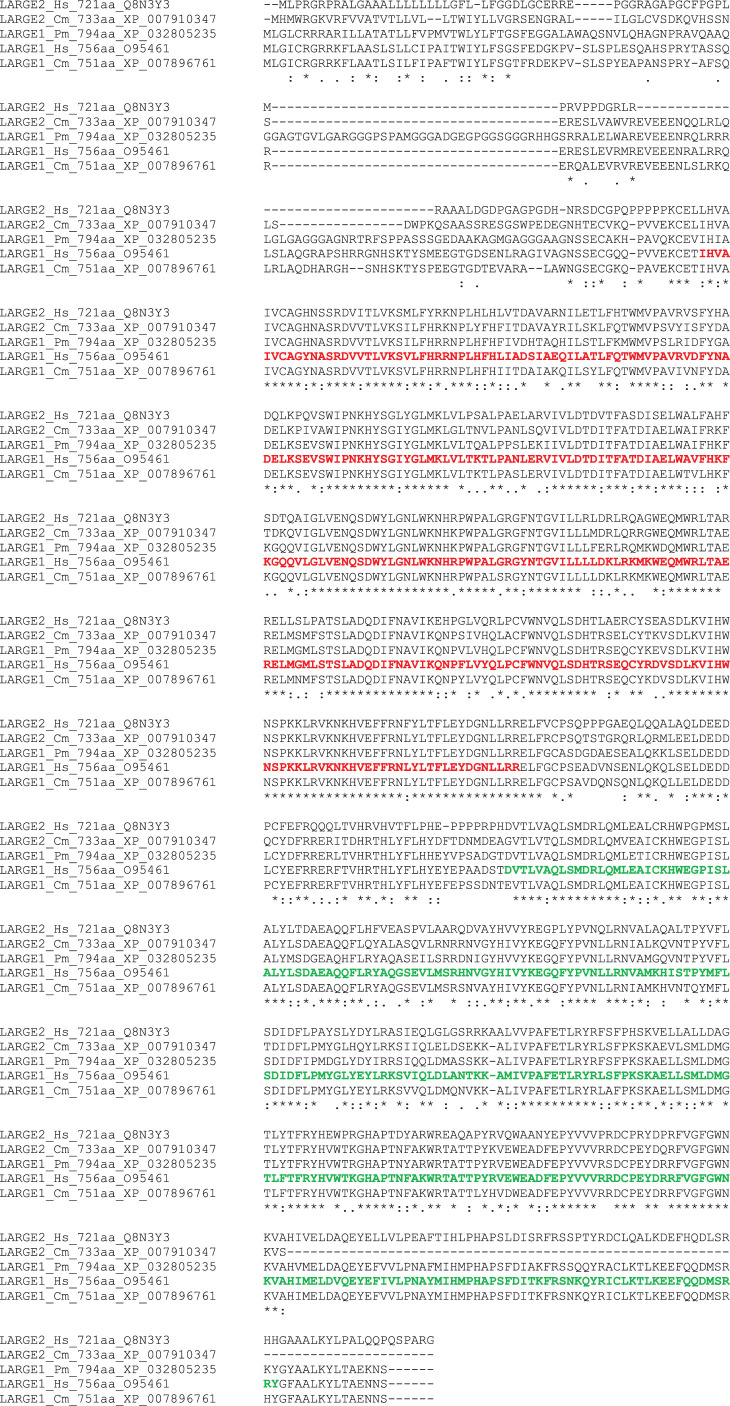
A multiple alignment of human LARGE1 and LARGE2 sequences with those from elephant shark (Chondrichthyes) and sea lamprey (Cyclostomata), showing the organization and conservation of the two subsequent catalytic domains oriented towards the Golgi lumen [70]. The two human LARGE1 catalytic domains are highlighted in red (xylosyltransferase domain) and green (glucuronyltransferase domain), respectively. Codes: Hs, *Homo sapiens*; Cm, *Callorhinchus milii* (elephant shark); Pm, *Petromyzon marinus* (lamprey). Asterisk: identical residues; colon: conserved substitutions; dot: semi-conserved substitutions.

LARGE1 must be seen as one of the most crucial enzymes of the cascade since it is responsible for the synthesis of matriglycan, whose tandemly repeated disaccharide units are those specifically involved in the recognition by the so-called LG (laminin globular) domains harboured by DG-binding partners [4]. LARGE2 is a paralogue of LARGE1 with a demonstrated narrower tissue distribution in mice and with no significant expression in skeletal muscle [70], which shows the same enzymatic properties as LARGE1 [96]. The alignment between LARGE1 and LARGE2 from different species reported in figure 3 reveals a very high degree of homology, in which the conservation of the modules corresponding to the xylosyltransferase and the glucuronic acid transferase domains separated by a linker is confirmed [67].

It was previously observed that only the LARGE1 isoform was present in invertebrates and suggested that a gene duplication took place in vertebrates [70]. Indeed, this is in line with the results of the current analysis, which identified a paralogue of LARGE1, corresponding to LARGE2, only in vertebrates, namely in Chondrichthyes (although only observed in *Callorhinchus milii*, elephant shark, figure 3), Teleostei, Amphibia, Reptilia, Aves and Mammalia but not in Cyclostomata. It remains unclear whether LARGE1 was indeed duplicated in all Chondrichthyes, since LARGE2 could not be found in other species (as for example *Carcharodon carcharias*, white shark). Notably, the presence of additional significant matches in *Ciona Intestinalis* and *Styela clava* might suggest duplication also in Urochordata (see electronic supplementary material).

It was previously shown that two paralogues of DG can be found in the lamprey (*Lethenteron japonicum*) [5], and this is further confirmed by the two DG matches (XP_032827644, 939 a.a. and XP_032822703, 848 a.a.) found in *Petrozon marinus* at NCBI. We have observed also two paralogues of DG in species belonging to the Acanthomorpha lineage [97]. Therefore, these are likely to represent isolated events possibly independent from overall genome duplications [5,6].

The real physiological relevance of LARGE2 remains unclear, as knockout of *LARGE2* in mice is phenotype-less [98], while knockout of *LARGE1* generates a severe phenotype [99], in line with the high expression of LARGE1 in brain, heart and skeletal muscle [70]. However, evidence collected on LARGE2 point to its importance in the kidney [98], in prostate cancer [100,101], and in human colonic epithelium and colorectal cancer [102]. In addition, it was shown that LARGE2 can modify proteoglycans with the laminin-binding glycan [103]. All these results point towards a specific significance of LARGE2 rather than a mere functional redundancy. Therefore, in Cyclostomata, *LARGE2* is likely to have undergone a selective inactivation, while in the other Chordata lineages the presence of a paralog gene is likely to have conferred an important degree of evolutionary advantage.

## 4. Conclusion

This initial reconstruction of the overall evolutionary pathway behind the rise of the M3 core structure and the DG/laminin axis implies that (i) all the enzymes of the M3 cascade have an ancestral origin that pre-dates DG and were already well consolidated before metazoan explosive rise; (ii) when DG came into play, at the level of basal Metazoa it was already able to hijack this pre-existing enzymatic machinery and prompt it to assemble into a specific DG-dedicated cascade starting with the initial *O*-mannosylation driven by POMT1/POMT2; (iii) the M3 cascade then enjoyed a prosperous evolutionary success in all the animal lineages, together with the DG/laminin axis it contributes to form. What was observed instead in Porifera and Nematoda is likely to represent examples of evolutionary divergence.

We believe that the high degree of conservation found in all the enzymes belonging to the M3 core structure cascade further stresses the importance of the enzymatic machinery responsible for DG sugar decoration and ultimately for the establishment of the DG-laminin axis, so fundamental to the achievement of a progressive muscle stability throughout all the Metazoan lineages. Such conservation might have conferred a significant evolutionary advantage, starting as early as from basal metazoans such as Placozoa and Cnidaria. This scenario fits with the idea of the evolutionary success of an integrated ‘molecular system’ of enzymes and their corresponding receptor-binding target system, able to accommodate new demanding biological, physiological and morphological requirements necessary for improving movement and locomotion skills (food collection, fighting, escaping from predators and so on).

## Data accessibility

The data that support the findings of this study are all available within the manuscript and the electronic supplementary material [104].
